# Characterization and functional prediction of the microRNAs differentially expressed in a mouse model of concanavalin A-induced autoimmune hepatitis

**DOI:** 10.7150/ijms.47766

**Published:** 2020-08-27

**Authors:** Yang Liu, Hao Chen, Jianheng Hao, Zhencheng Li, Tiezheng Hou, Huiqin Hao

**Affiliations:** 1 College of Basic Medical Sciences, Shanxi University of Chinese Medicine, Jinzhong, 030619, PR China.; 2 Basic Laboratory of Integrated Traditional Chinese and Western Medicine, Shanxi University of Chinese Medicine, Jinzhong, 030619, PR China.

**Keywords:** autoimmune hepatitis, concanavalin A, microRNA, microarray, Gene Ontology, KEGG

## Abstract

In order to investigate the altered expression of microRNAs (miRNAs) in the development of autoimmune hepatitis (AIH), the aberrantly expressed miRNAs in the concanavalin A (Con A)-induced AIH mouse model were identified for the first time with microarray in this study. A total of 49 miRNAs (31 up- and 18 down-regulated) were screened out, and the qRT-PCR validation results of 12 chosen miRNAs were consistent with the microarray data. Combined with the profiling of differently expressed mRNAs in the same model (data not shown), 959 predicted target genes (601 for up- and 358 for down-regulated miRNAs) were obtained according to the intersection of databases miRWalk and miRDB, and several hub genes were obtained from the regulatory networks, including *Cadm1* and* Mier3*. These target genes were significantly enriched in the Gene ontology (GO) terms of “transcription, DNA-templated”, and were annotated in 47 signaling pathways, comprising “Wnt signaling pathway”, “Hippo signaling pathway”, “Ferroptosis” and “mitogen-activated protein kinase (MAPK) signaling pathway”, according to the GO and Kyoto Encyclopedia of Genes and Genomes (KEGG) enrichment analysis. In the miRNA-GO-network, mmu-miR-193b-3p were exhibited in 33 GO terms of biological processes (BP), and the most significantly regulated GO term in BP categories was “regulation of transcription, DNA-templated”. While in the miRNA-pathway-network, mmu-miR-7005-5p were enriched in 37 pathways, which was more than the other specifically expressed miRNAs, and the most significantly enriched pathways were “Endocytosis” and “MAPK signaling pathway”. In conclusion, these differently expressed miRNAs seemed to be associated with the onset of AIH, and have the potential to serve as the new targets on the treatment of this disease.

## Introduction

As one type of classical autoimmune disease, autoimmune hepatitis (AIH) occurs at any age and in any ethnicity, and shows the female preponderance with a female-to-male ratio of 4:1 1, 2. This disease features histologically by interface hepatitis and lymphocytic infiltration in liver, and is distinguished serologically by the presence of autoantibodies and high levels of immunoglobulin G (IgG), alanine aminotransferase (ALT), aspartate aminotransferase (AST) 3, 4. The annual incidence (ranges between 0.67 and 2.23 cases per 100,000 persons) and prevalence (varies from 2.4 to 42.9 cases per 100,000 persons) of AIH are diverse depending on the geographical location 5-9, and the etiology of this disorder is likely to be related with the genetic (e.g. allelic variants of genes within the human leukocyte antigen region on the short arm of chromosome 6) and environmental factors (comprising viruses, bacteria, alcohol and deficiency of vitamin D) 10, 11. The immunologic disarrangement of AIH is thought to be based on the sustained response to self-antigens within the liver provoked or mediated by the combinations of pathogenic factors, and it is characterized by T-lymphocyte mediated disorganization (predominated by CD4+T cells) and imbalance in the regulation of immune cells (e.g. decrease in the number of Tregs) 12-14. Nevertheless, the exact pathogenesis of AIH has not yet been illustrated.

MicroRNA (miRNA), produced by two types of RNase named Drosha and Dicer, is a major class of non-coding RNA molecule with ~22 nucleotides in length, and plays an important role in regulating gene expression negatively at the messenger RNA (mRNA) level 15. The mRNA silencing functions of miRNAs (i.e. translational repression, mRNA decay and mRNA deadenylation) are achieved by base pairing with its target mRNAs, and the Argonaut proteins (AGO) act as the effectors 16. The domain at the 5' end of miRNAs (nucleotides 2-8), termed as “miRNA seed”, is crucial for target recognition 17. The miRNA-binding sites are mainly located in the 3' untranslated region (UTR) of target mRNAs, and at least one conserved miRNA-binding site can be found in more than 60% of human protein-coding genes. Furthermore, most protein-coding genes are under the control of more than one miRNA in consideration of abundant non-conserved binding-sites also exist 18. Therefore, it is not unexpected that the abnormal expression of miRNAs is tightly linked to many human diseases (particularly in cancer), and the aberrantly expressed miRNAs are being pursued as new clinical diagnostic and therapeutic targets 19, 20.

So far, a number of miRNAs have been proved to be the important negative regulators taking part in the development of AIH 21-25. However, the exact changes of miRNA expression profiling in AIH have not been reported. The differentially expressed miRNAs in a mouse model of concanavalin A (Con A)-induced AIH were both screened using microarray chip and systematically analyzed with bioinformatic methods for the first time herein, in order not only to evaluate the latent roles of miRNAs in the etiopathogenesis of AIH, but also to offer new therapeutic strategy for this worldwide hepatitis.

## Materials and Methods

### Ethics Statement

Specific pathogen free (SPF) grade C57BL/6 mice (male, 6 weeks old, 20-22 g) were obtained from Vital River Laboratory Animal Technology Co., Ltd. (Beijing, China). The mice were housed under controlled temperature (21-24 °C) and humidity (40%-60%), 12 h dark/light cycles and feeding ad libitum for a week before the study. The animal experiments complied with the rules of National Institutes of Health guide for the care and use of Laboratory animals (NIH Publications no. 8023, revised 1978), and approved by the Ethics Committee of Shanxi University of Chinese Medicine (Permit no. 2019LL41).

### Reagents and chemicals

Con A was purchased from Solarbio Science & Technology Co., Ltd. (Beijing, China), batch no. C8110. Chloral hydrate and UNlQ-10 Column Total RNA Isolation Kit were gained from Sangon Biotech Co., Ltd. (Shanghai, China), batch number: A600288 and B511321. Maxima Reverse Transcriptase was gotten from Thermo Fisher Scientific (China) Co., Ltd. (Shanghai, China), batch no. EP0743.

### Animal experiment

The mice in model group (n=4) and sham group (n=4) were administrated Con A solution (15 mg/kg, dissolved in pyrogen-free saline) or pyrogen-free saline via tail vein, severally. All mice were anesthetized to death at 8 h after administration, and liver tissues were harvested under low temperature and sterile conditions. The integrity of the total RNA extracted from these hepatic tissue samples were assessed with Agilent Bioanalyzer 2100 (Agilent Technologies, USA).

### Microarray hybridization and data analyses

The Agilent Mouse miRNA Microarray Kit (Design ID: 070155, release 21.0, 8x60K, containing 1 902 probes for mature miRNA) was implemented in our research. The experimental operations of sample labeling, microarray hybridization and washing were performed based on the manufacturer's standard protocols. After washing, the arrays were scanned with the Agilent Scanner G2505C (Agilent Technologies, USA). Feature Extraction software (version10.7.1.1, Agilent Technologies, USA) and Genespring software (version 14.8, Agilent Technologies, USA) were employed to accomplish the basic analysis with the raw data by normalizing the data with the quantile algorithm.The standardized data were filtered for subsequent analysis under the conditions that at least 75% of the samples labeled as “detected” were met. Pearson Correlation and principal component analysis (PCA) were implemented to analyze the correlation between different samples so as to evaluate the reproducibility of the data. Then, differentially expressed miRNAs were identified through the fold change (FC) as well as *P* value calculated using t-test. The threshold set for up- and down-regulated miRNAs were |FC| ≥2.0 and *P* value < 0.05. Hierarchical clustering analysis was also carried out to show the distinguishable expression pattern of miRNAs among different samples.

### Validation of quantitative real-time polymerase chain reaction (qRT-PCR)

Twelve differentially expressed miRNAs (six up- and six down-regulated) were picked up for qRT-PCR amplification to validate the microarray results. Step One PULS real-time fluorescent quantitative PCR (ABI, Foster, CA, USA) was utilized for amplification. Features of the chosen miRNAs were presented in **Table 1.** The primers were synthesized by Sangon Biotech Co., Ltd. (Shanghai, China), and their sequences were listed in **Table 2.** The expression levels of miRNAs were normalized to *U6* and were calculated with the 2^-ΔΔCt^ method 26.

### Prediction of miRNA targets

In order to explore the potential functions of these screened differentially expressed miRNAs, combined with the results of differentially expressed mRNAs obtained in the same model by us (data not shown), the predicted target-gene for these miRNAs was filtered out according to the intersection of miRWalk database (Version 3.0) 27 and miRDB database (release date: June, 2019) 28 by using the VENNY tool 29. In this study, only the predicted target genes with prediction score > 80 (in miRDB) and binding *P* value = 1 (in miRWalk) were taken into the further analysis. The miRNA-mRNA interaction networks were constructed and visualized with Cytoscape software (version 3.7.2, http://cytoscape.org/).

### Functional annotation analyses of the predicted target genes

Enrichment analysis of Gene ontology (GO) [30]and Kyoto Encyclopedia of Genes and Genomes (KEGG) (Release 85.0, January 1, 2018,) were applied to determine the biological functions of these target genes. All the predicted genes were annotated with their GO information, including cellular components (CC), molecular functions (MF) and biological processes (BP). Lower *P* values indicated more significant enrichment of the target genes in these Go terms. The false discovery rate (FDR), utilized for multiple testing corrections of raw *P*-value, was set as the cutoff for selecting significantly enriched functional GO terms (FDR ≤ 0.05). For KEGG pathway analysis, a pathway was significantly enriched only if it passed the count threshold, and the recommended threshold was *P* value ≤ 0.05. Lower *P* value represented higher correlation between the pathway and target genes.

### Statistical analysis

Independent -sample t-test was used to identify differentially expressed miRNAs with SPSS 25.0 software (SPSS Inc., Chicago, IL, USA). Statistical significance was considered as *P* value < 0.05.

## Results

### Identification and validation

**Figure 1:**
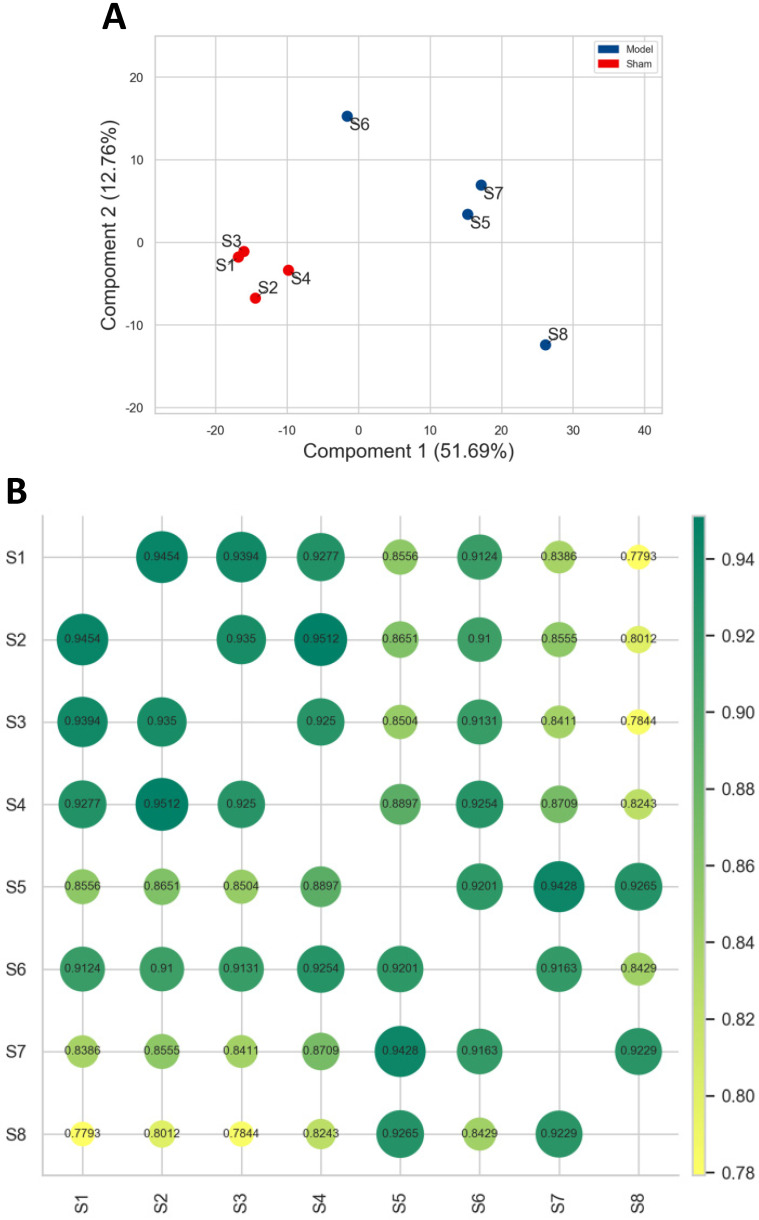
** Correlation analysis between different samples. A. **PCA. The blue and red dot represented the samples obtained in model and sham group. The shorter the distance between samples in same group, and farther away from the samples in different groups, that indicated the more the experimental reliability and rationality of sample selection. **B.** Pearson correlation analysis. Each dot represented the correlation between different samples, and the decimal in the dot was the coefficient of association. The larger the number, the greener and bigger the dot, the higher the relevance.

As shown in **Figure 1**, individuals in model group flocked together and separated significantly from the sham group, and the normalized data of each sample in sham group was with a very high correlation, and the same for the model group. It was indicated that the reproducibility of the data and experimental reliability satisfied the conditions for further analysis.

**Figure 2:**
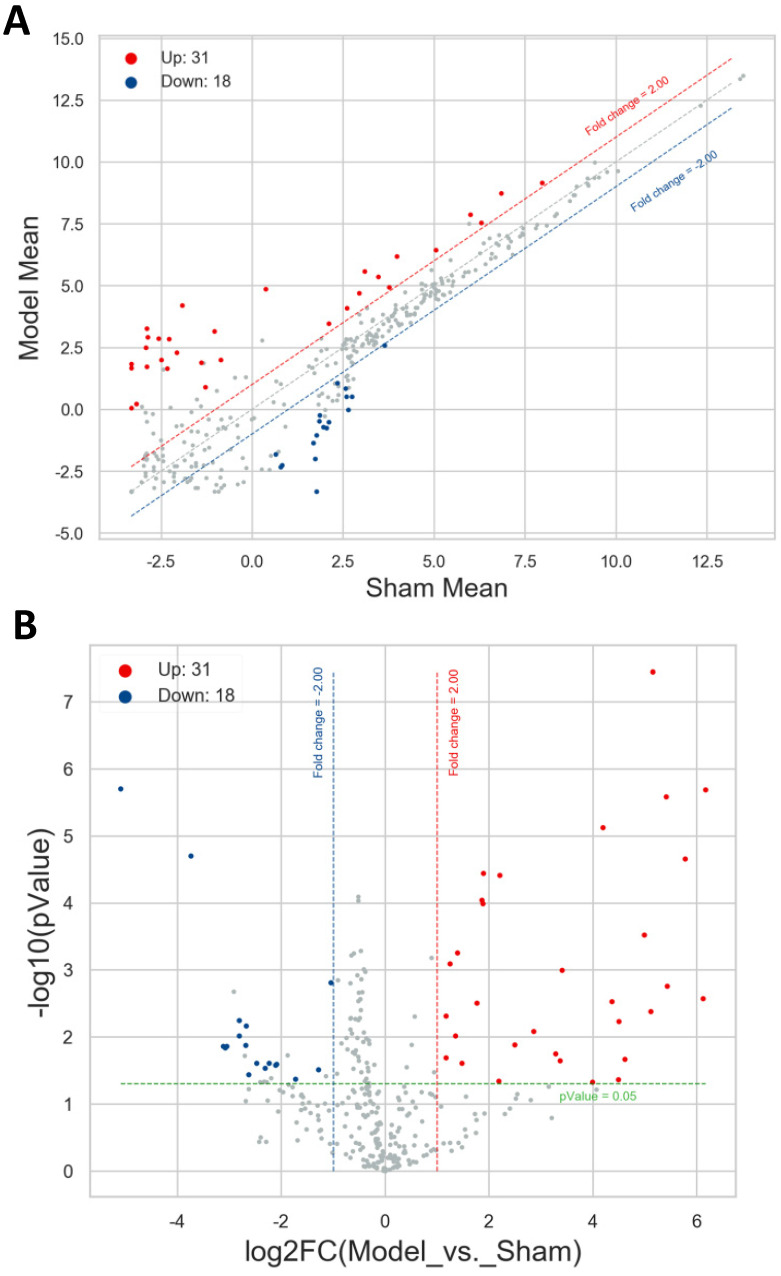
** Distribution of the differentially expressed miRNAs.** Red and blue points represented the up- and down-regulated miRNAs, gray points highlighted the miRNAs expressed not meet the threshold. Red and blue dotted line reflected the up- and down-expressed threshold (FC=2.0). Green dotted line denoted *P* value =0.05. **A.** Scatter plot. Horizontal line and vertical line signified the average expression level of each miRNA in sham group and model group. **B.** Volcano plot. Horizontal line and vertical line corresponded to log_2_FC and -log10(*P* value).

A total of 49 differentially expressed miRNAs were screened out with the threshold of |FC| ≥ 2.0 and *P* value < 0.05 between the model group and sham group, comprising 31 up- and 18 down-regulated miRNAs. These miRNAs were itemized in **Table S1**, and the most significantly up- and down-regulated miRNAs were mmu-miR-3091-5p (FC = 71.90) and mmu-miR-1927 (FC = -34.06). Then the scatter plot and volcano plot were utilized to make it comprehensible for the distribution results of these differentially expressed miRNAs. As exhibited in **Figure 2A and 2B**, the filtered-out miRNAs (red points for up- and blue points for down-regulated) were distinctly separated from the miRNAs not met the threshold (gray points), which manifested the screening results and were reliable. The more the miRNAs far away from the threshold (red or blue dotted line), the more closely it was related to the development of AIH.

**Figure 3:**
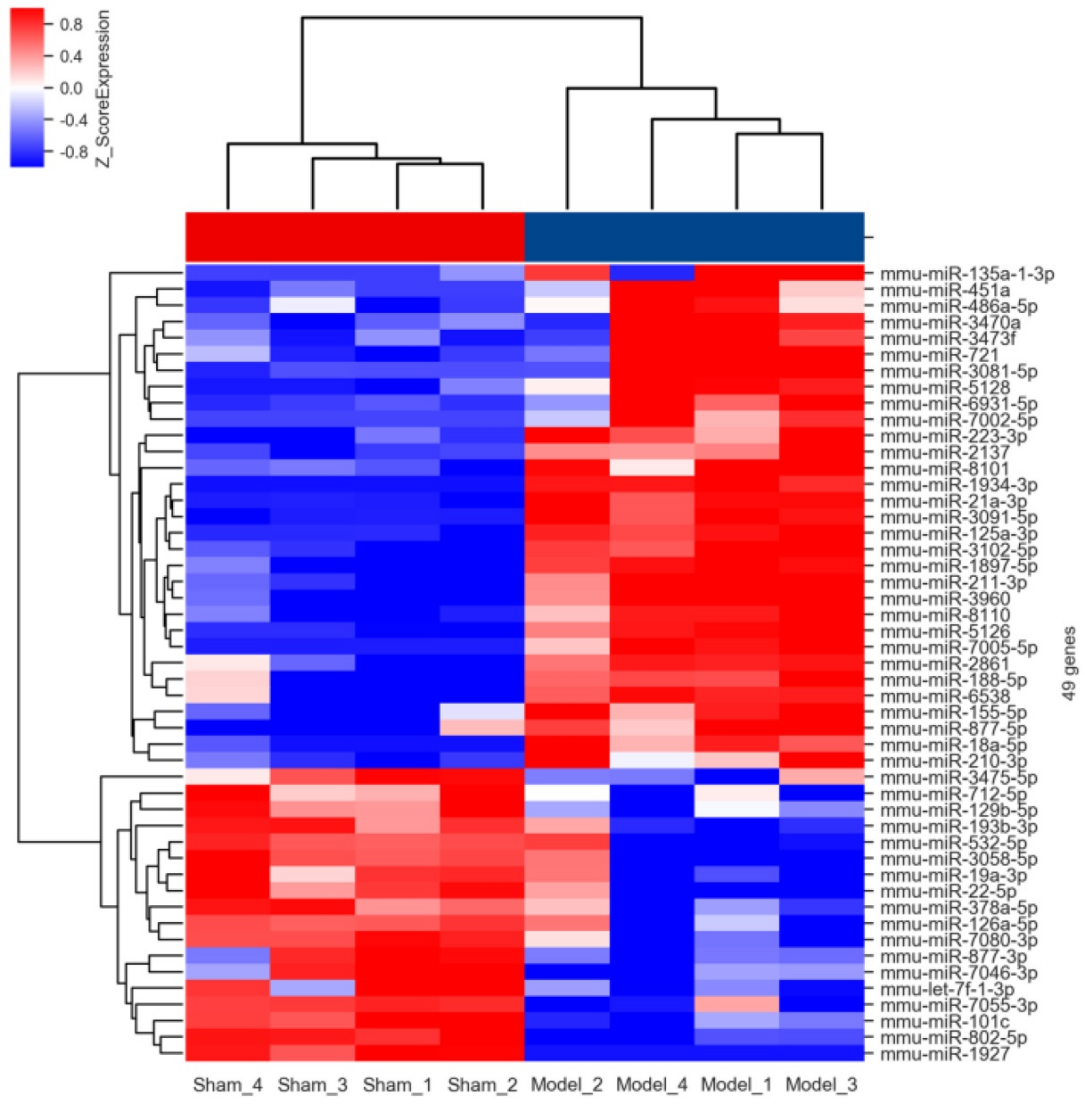
** Hierarchical clustering analysis of the differentially expressed miRNAs.** Each column in the heatmap of hierarchical cluster analysis indicated an individual sample (Sham 1-4 and Model 1-4), and each row represented an individual miRNA. The red and blue shades signified the expression levels of miRNAs above and below the relative expression among all samples.

The results of hierarchical clustering analysis based on the normalized expression values of up- (red shades) and down-regulated (blue shades) miRNAs were shown in **Figure 3** in the form of heatmap. It was obviously that these differentially expressed miRNAs possessed a discriminatory power to stratify AIH model from sham individuals, because all mice in the model group were clustered together and separated from the control subjects.

**Figure 4:**
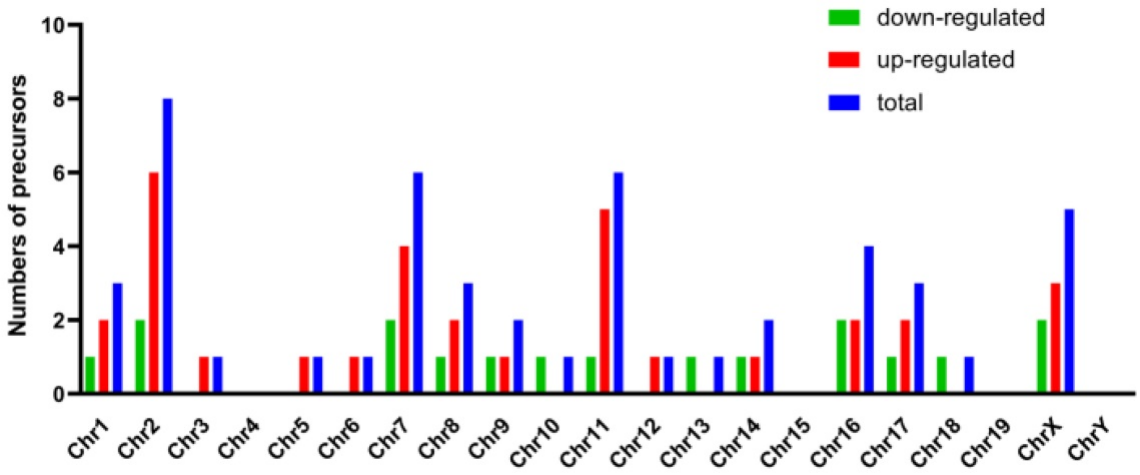
** Genomic locations of the precursors of these differentially expressed miRNAs.** Horizontal and vertical line represented the numbers of precursors and chromosome. Red, green and blue column separately indicated the numbers of precursors of up-regulated, down-regulated and total miRNAs.

Genomic locations of the precursors of these differentially expressed miRNAs were displayed in **Figure 4.** These precursors distributed on all chromosomes except chromosome 4, 15, 19 and Y. The number of precursors (8 of 49 precursors) expressed on chromosome 2 was the largest. It was in accordance with results of the location of the differentially expressed long non-coding RNAs (lncRNA) and differentially expressed mRNAs in the same model (data not shown), which indicated that the chromosome 2 was the most severely affected chromosome in the process of AIH.

**Figure 5:**
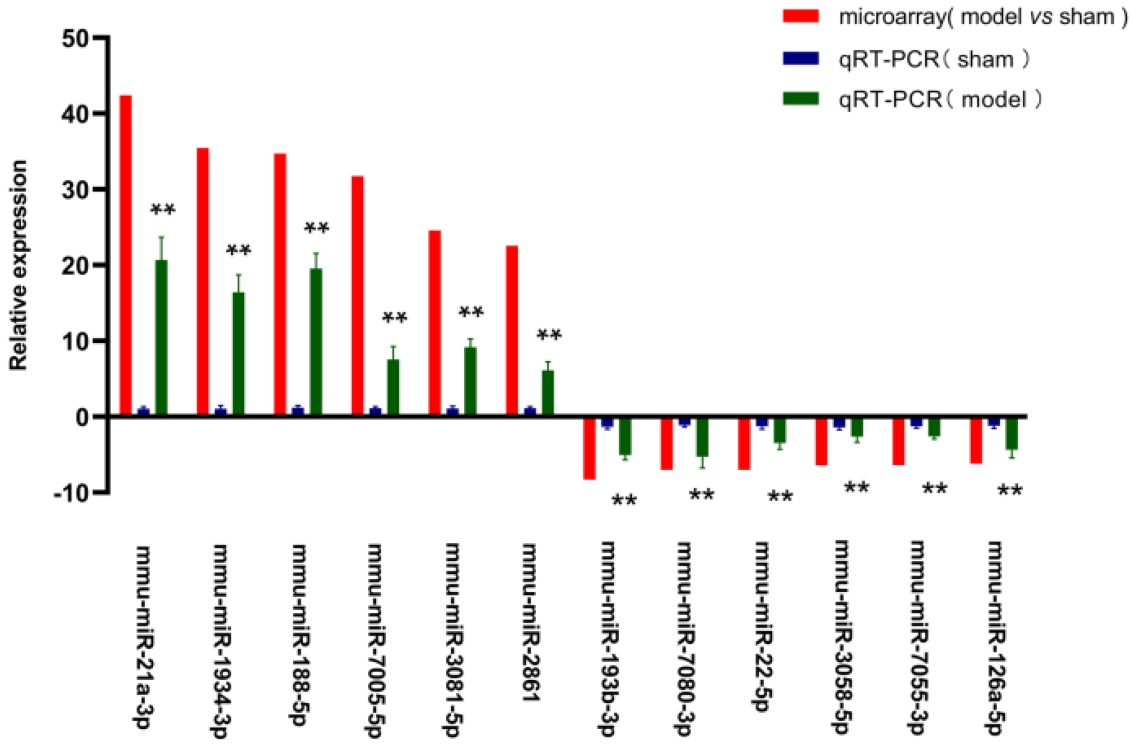
** QRT-PCR validation of the chosen miRNAs.** Y axis signified the relative expression level of the chosen miRNA in model group (n=4) compared with that in sham group (n=4), and the negative values indicated expression of the gene in model group was down-regulated. Red column represented the results of microarray (the data were normalised to the negative probe), while blue and green column reflected the findings in sham and model group with qRT-PCR (the data were normalised to *U6*). The data were shown with the mean±SD (error bars). All data in Figure 5 were statistically analyzed by Student's t-test and and *P* < 0.05 was considered statistically significant (**P value < 0.05 between model group and sham group).

For validating the microarray data, we chose twelve screened miRNAs (six up- and six down-regulated) to amplify with qRT-PCR assay. The relative expression of these 12 picked-up miRNAs was laid out in **Figure 5.** The expression level of *mmu-miR-21a-3p* (20.69- fold) was the most elevated, followed by *mmu-miR-188-5p* (19.54-fold), *mmu-miR-1934-3p* (16.40-fold), *mmu-miR-3081-5p* (9.17-fold), *mmu-miR-7005-5p* (7.56-fold), *mmu-miR-2861* (6.12-fold), whereas *mmu-miR-7080-3p* (5.28-fold) was the most down-regulated miRNAs, and the next were *mmu-miR-193b-3p* (5.04-fold),* mmu-miR-126a-5p* (4.34-fold), *mmu-miR-22-5p* (3.49-fold), *mmu-miR-3058-5p* (2.65-fold), *mmu-miR-7055-3p* (2.61-fold) in turn. The trends on the expression changes detected with qRT-PCR were consistent with microarray data, which indicated that the credibility of the microarray results was confirmed, as well as it was met the conditions for the further analysis.

### Target genes prediction

**Figure 6:**
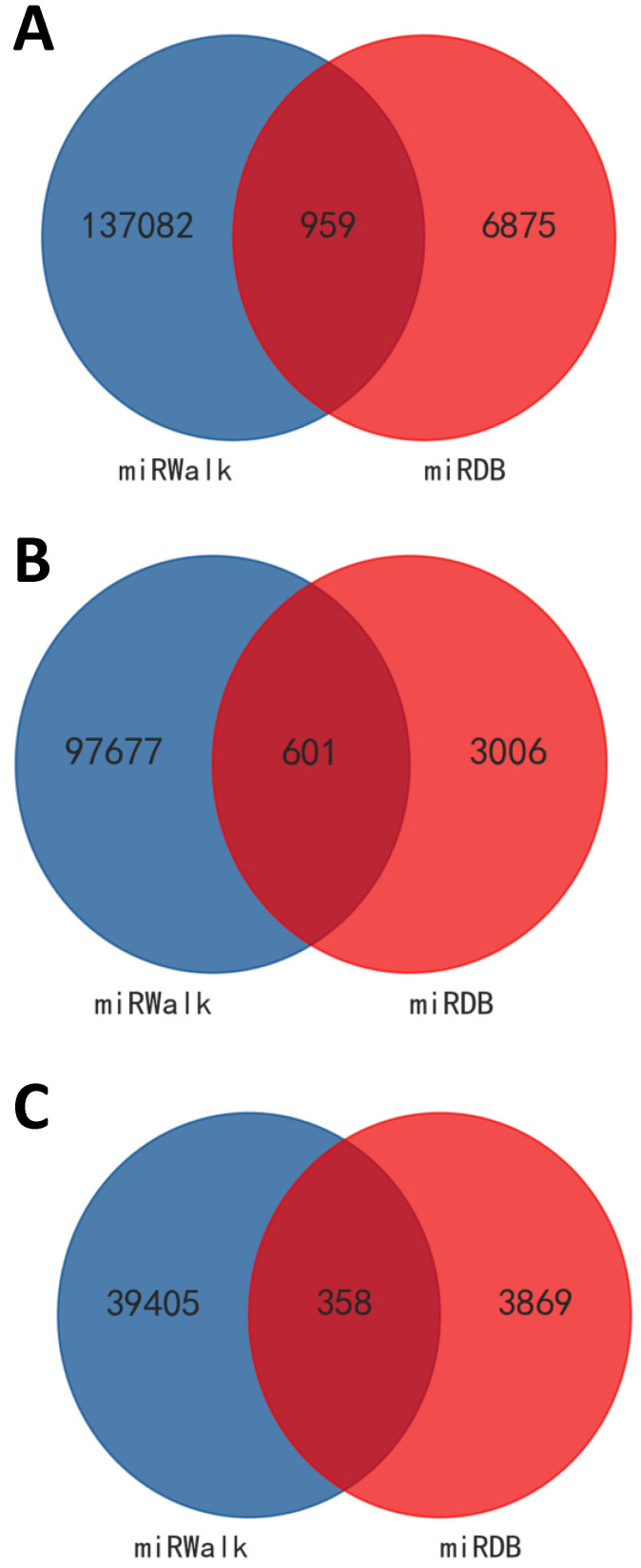
** Predicted target genes for these differentially expressed miRNAs.** Targets genes were obtained according to both the screened results of differently expressed mRNAs (data not shown) and the intersection of miRDB and miRWalk**.** VENNY tool was used to visualize the results. A: for all the differentially expressed miRNAs. B: for up-regulated miRNAs. C: for down-regulated miRNAs.

**Figure 7:**
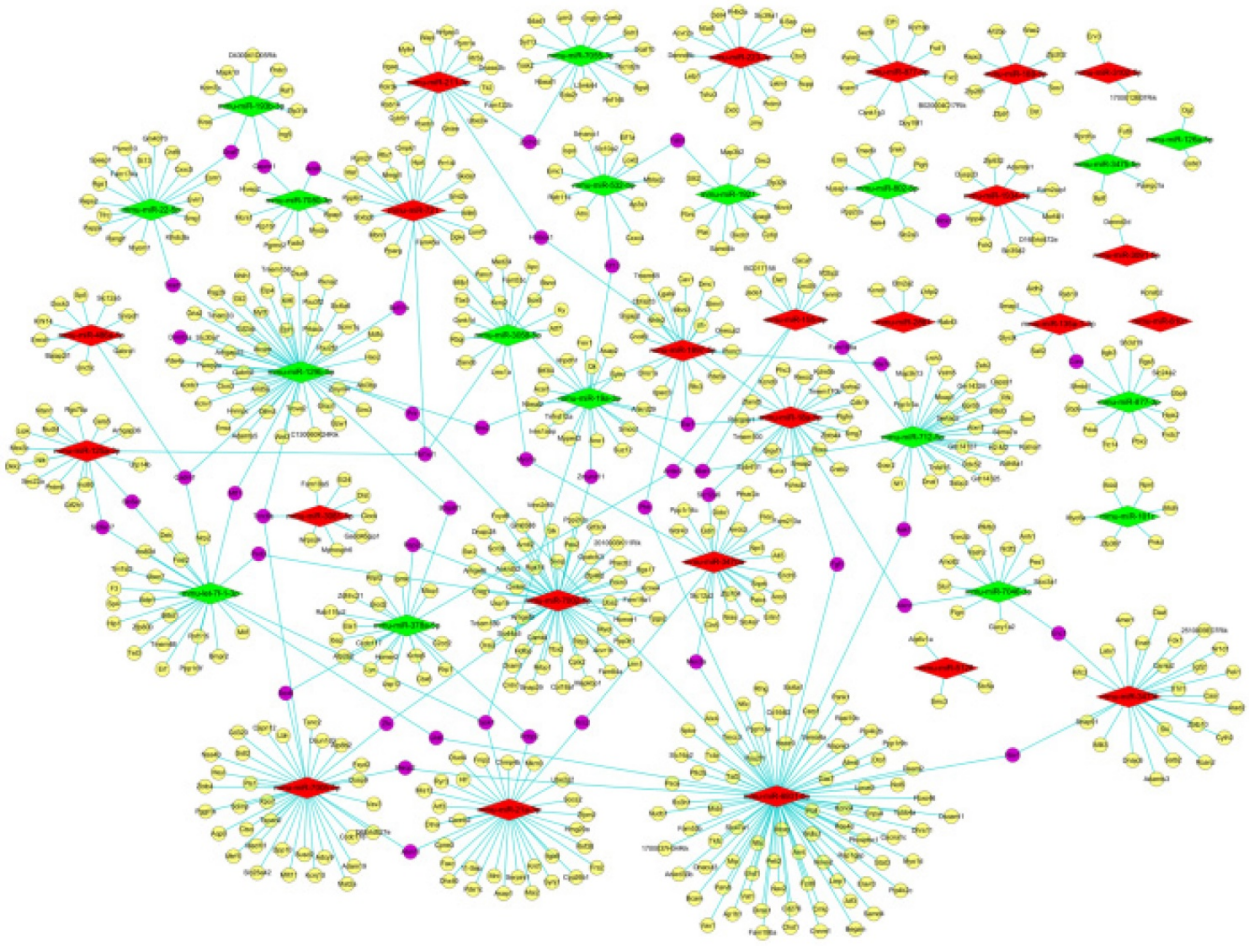
** The miRNA-mRNA networks of these differentially expressed miRNAs.** Red and yellow diamond represented the up- and down-regulated miRNAs. Yellow ellipse nodes indicated the predicted target genes, purple ellipse nodes signified the hub genes. The edges meant the regulatory relationships between the miRNA and target genes.

As appeared in **Figure 6** with Venn diagrams, based on both the screened results of differently expressed mRNAs (data not shown) and the intersection of miRDB and miRWalk, 959 target genes were obtained for these 49 differentially expressed miRNAs (601 genes for the 31 up- and 358 genes for the 18 down-regulated miRNAs) by the aid of bioinformatic methods. According to the intricate miRNA-mRNA networks constructed and visualized in **Figure 7**, the possible regulatory functions of these screened miRNAs were revealed. It was apparent that the modulation mechanisms of these differentially expressed miRNAs were elusive because each miRNA targeted on more than one mRNA, vice versa for several mRNAs (named as hub gene). Except the differentially expressed miRNAs, the hub genes obtained from the regulatory networks, including *Cadm1* and* Mier3*, were also the new biomarkers needed to research thoroughly in our future study on the pathogenesis of AIH.

### GO enrichment analysis

**Figure 8:**
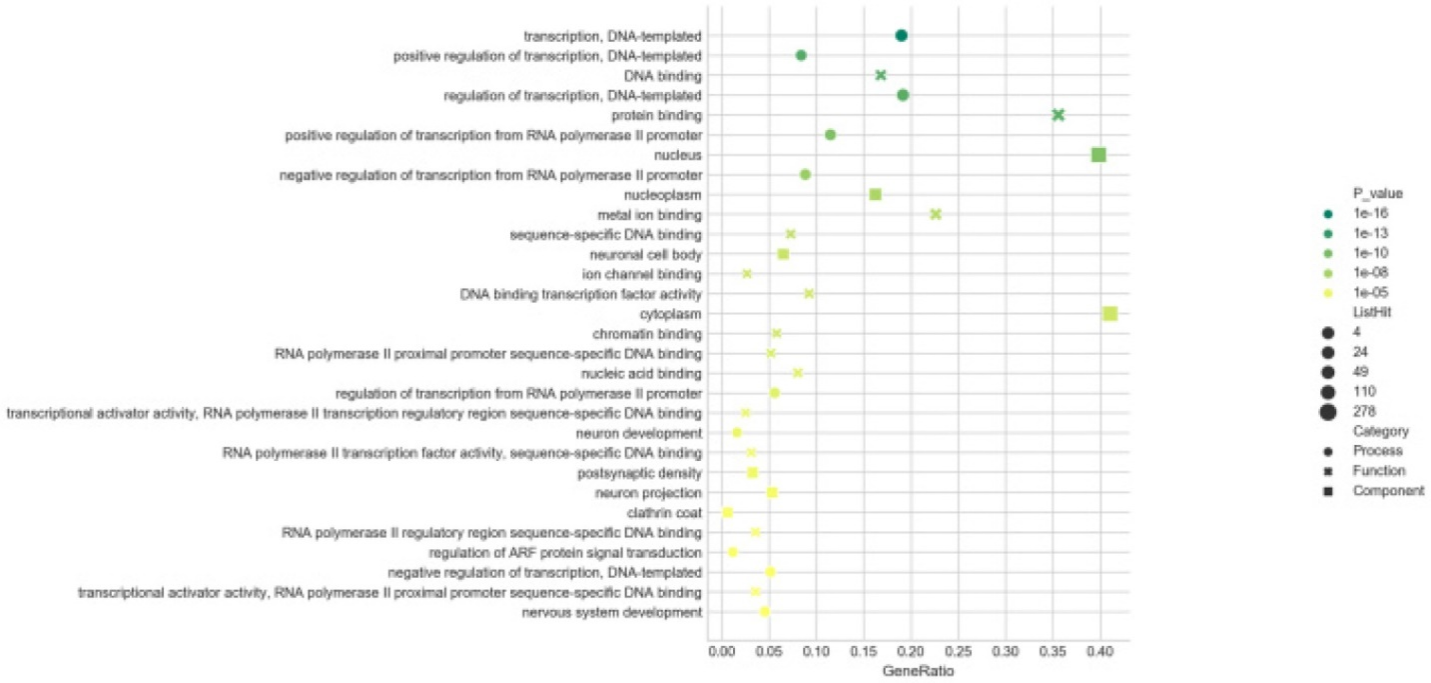
**GO enrichment analysis.** Bubble diagrams were drawn based on the top30 GO terms listed in ascending order by *P*-value. Axis of ordinates and abscissa represented GO terms and degree of enrichment (gene ratio). Different shapes of the dots correspond to different GO categories (●:biological processes, **×**: molecular function, █: cellular components). The larger the dot, the more genes that enriched in this GO terms. The greener the dot, the higher the enrichment significance.

To better explore the potential roles of these differentially expressed miRNAs played in the etiopathogenesis of AIH, the predicted target genes were submitted to the GO database for performing functional annotation. There were 679, 678 and 672 target genes annotated in the categories of BP, CC and MF, respectively. With the threshold of FDR ≤ 0.05, the numbers of GO terms classified in BP, CC and MF were 20, 21 and 43, severally. Results of GO analysis evinced that the target gens of all screened miRNAs were mostly response to the terms of “transcription, DNA-templated” (BP), “protein binding” (MF) and “nucleus” (CC) (in **Figure 8**). It was shown that these miRNAs played a vital role in the process of transcription. Moreover, 601 target genes potentially modulated by the 31 up-regulated miRNAs were annotated in 33 GO terms (10 in BP, 7 in CC, and 16 in MF), and 358 target genes for the 18 down-regulated miRNAs were related to 37 GO terms (14 in BP, 5 in CC, and 18 in MF), in accordance with the threshold of FDR ≤ 0.05. The top30 (in ascending sort order of *P* value) GO terms were exhibited in **Table S2 and S3.** The target genes of up- and down-regulated miRNAs were all also obviously enriched in the GO terms of “transcription, DNA-templated” (BP), “DNA binding” (MF) and “nucleus” (CC). This result suggested that the expression balance between these differentially expressed miRNAs was crucial for modulating the genes transcription during the occurrence of AIH.

**Figure 9:**
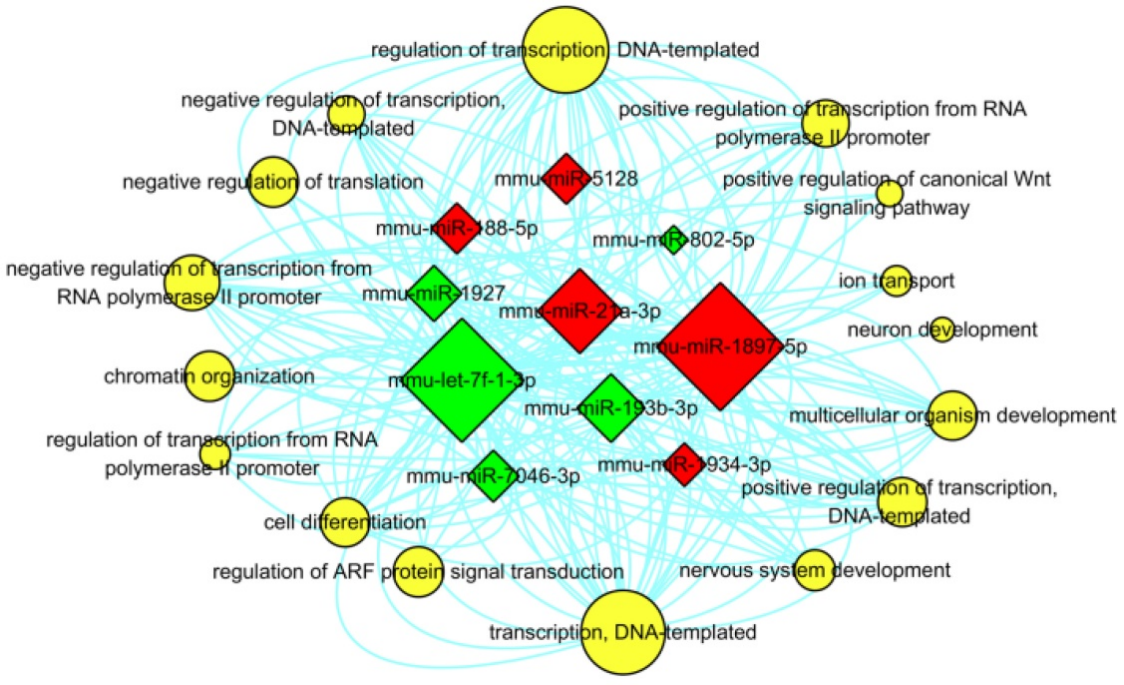
** Interaction between the annotated GO terms of BP and the top 5 up- and down- regulated miRNAs.** The red and green square represented up- and down regulated miRNAs. The yellow circular nodes indicated the target GO terms of BP. The lines notified the interaction relationships between miRNAs and target biological processes. The size of square nodes and yellow circular nodes reflected the degree to which the miRNAs and target GO terms contributed to the network.

In addition, in order to determine the regulatory associations between the key miRNAs and hub GO terms, the miRNA-GO-network was constructed for the top 5 (in descending sort order of |FC|) up- and down-regulated miRNAs which were annotated in the GO categories of BP with the threshold of FDR ≤ 0.05 (shown in **Figure 9**). In this network, mmu-miR-193b-3p was exhibited in 33 GO terms of BP, which contributed more than the other specifically expressed miRNAs to the network. The most significantly regulated GO term in BP categories was “regulation of transcription, DNA-templated” (GO: 0006355), defined as any process that modulates the frequency, rate, or extent of cellular DNA-templated transcription.

### KEGG enrichment analysis

**Figure 10:**
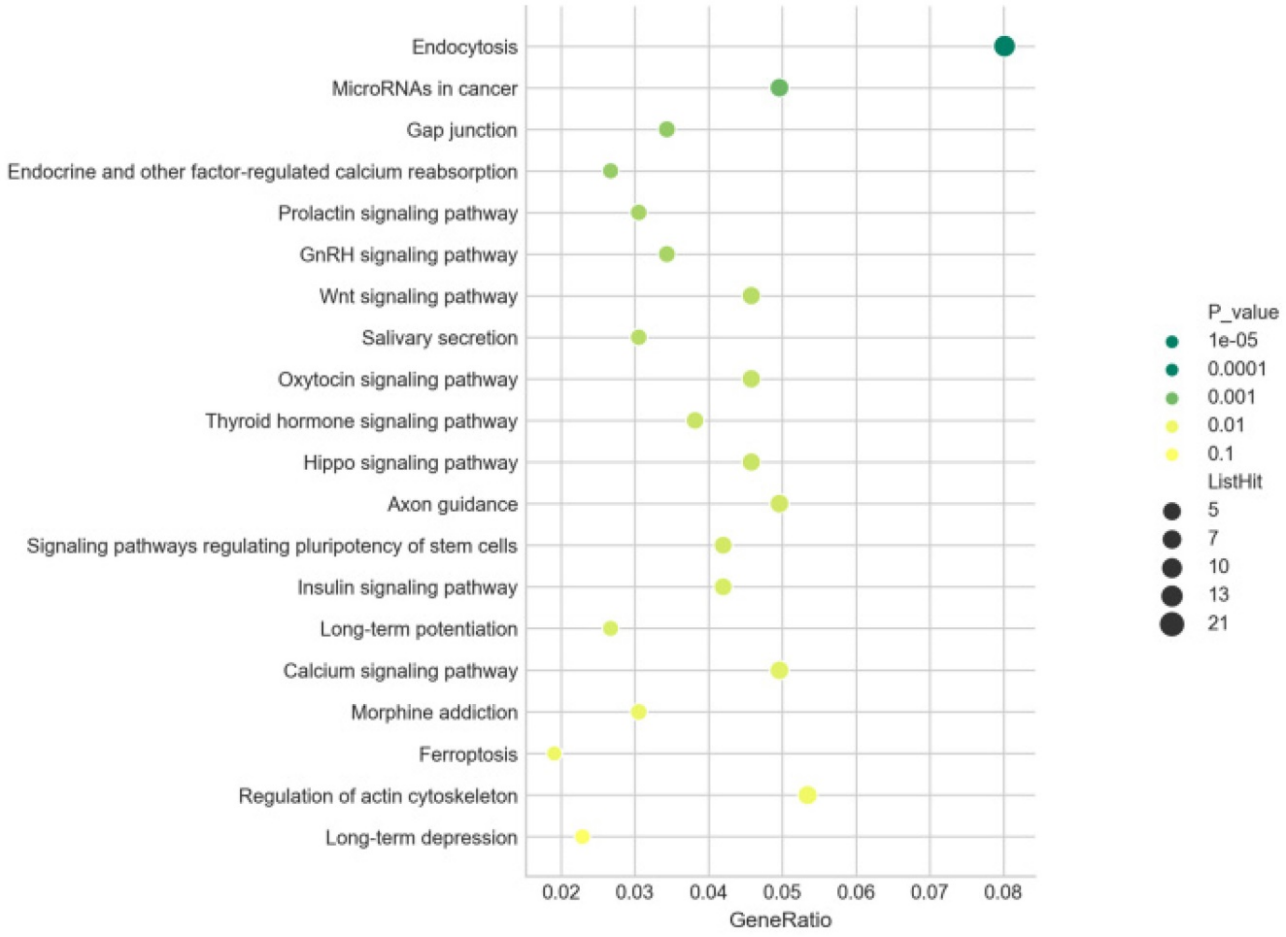
** KEGG enrichment analysis.** Bubble diagrams were drawn based on the top20 pathways in ascending sort order of *P* value. Axis of ordinates and abscissa represented pathways and degree of enrichment (gene ratio), severally. The larger the dot, the more genes that enriched in this pathway. The greener the dot, the higher the enrichment significance.

There were 262 of 959 predicted target genes annotated in 47 KEGG pathways for all differentially expressed miRNAs with the *P* value ≤ 0.05. The top 20 pathways ranked in ascending order by *P*-value were listed in **Figure 10**, including “Wnt signaling pathway” (path: mmu04310), “Hippo signaling pathway” (path: mmu04390), and “Ferroptosis” (path: mmu04216). The most significantly enriched pathway was “Endocytosis” (path: mmu04144). These results revealed the annotated pathways were the key for the differentially expressed miRNAs involving in the development of AIH. Moreover, our findings also pointed 20 KEGG pathways for the up- (171 of 601 predicted target genes enriched) and 26 KEGG pathways for the down-regulated miRNAs (104 of 385 forecast target genes enriched) with the *P* value ≤ 0.05. The top 20 pathways ranked in ascending order by *P*-value were also shown in **Table S4 and S5**, including some common pathways, such as “Wnt signaling pathway”. It was signified that the modification of these common signaling pathways were resulted from coaction of these screened miRNAs.

**Figure 11:**
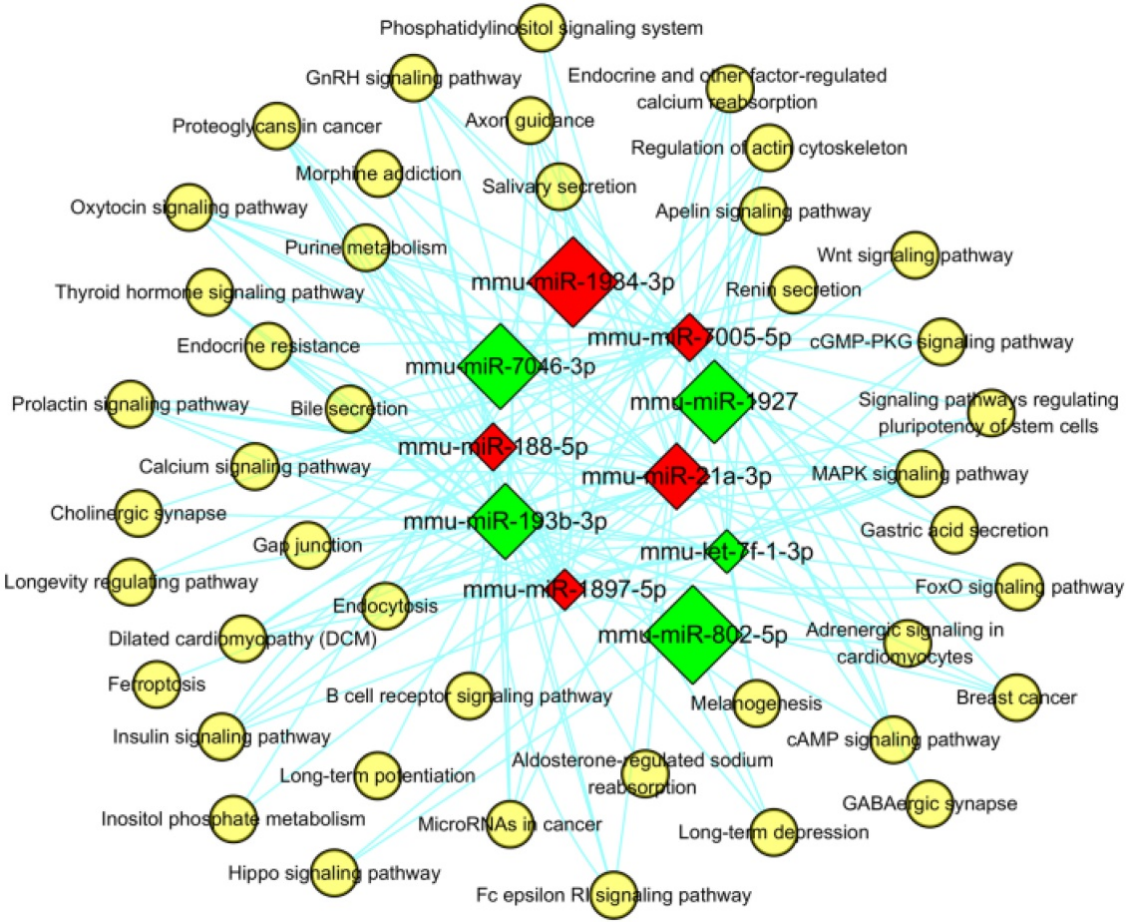
** MiRNA-pathway network for the top 5 up- and down-regulated miRNAs.** The red and green square represented up- and down regulated miRNAs. The yellow circular nodes indicated the enriched pathways. The lines reflected the interaction relationships between miRNAs and pathways. The size of square nodes signified the degree to which the miRNAs contributed to the network.

Furthermore, the miRNA-pathway-network was also constructed for the top 5 (in descending sort order of |FC|) up- and down-regulated miRNAs which were enriched in the KEGG signaling pathways with the threshold of *P*< 0.05 (shown in **Figure 11**), in order to determine the regulatory relationships between the key miRNAs and hub pathways. In this network, mmu-miR-7005-5p were enriched in 37 pathways, which was more than the other specifically expressed miRNAs. The most significantly enriched pathways were “Endocytosis” and “mitogen-activated protein kinase (MAPK) signaling pathway” (path: mmu04010).

## Discussion

With the progress of researches on miRNAs, it is thought that miRNAs are related to a series of biological processes, and their abnormal expression has been described in lots of autoimmune diseases, including AIH 31-33. As a kind of high-throughput detection technique, miRNA microarray is supposed to be a powerful tool to detect a set of differentially expressed miRNAs among different samples concurrently, and make it available to uncover numerous signaling pathways possibly dysregulated in the pathogenesis of many diseases if combining with the bioinformatic analysis 34, 35. The aberrantly expressed circulating miRNAs in type 1 AIH patients have been identified with microarray analysis, and the circulating miR-21 and miR-122 were not only found to be involved in the mediation of inflammatory processes, but also regarded as the potential biomarkers for treatment on this disease 36. Because the peripheral blood is easier to acquire and can provide a large biosensor pool in the form of gene transcripts, so it is deemed to be an ideal surrogate tissue for the diagnosis and prognosis of diseases. However, the application of peripheral blood cells and serum miRNAs profiling in the mechanism research on AIH has its limits, for the reason that just an estimated 80% of all human genes are expressed in peripheral blood cells 37. Moreover, it is unable to normalize the content of serum miRNAs with a reliable housekeeping miRNA, as the exact release mechanism of miRNA into circulation is yet to be defined and the impact of factors that regulate the expression of circulating miRNAs are also unknown 38. Hence, just screening the circulating miRNA-based biomarkers cannot meet the needs of the comprehensive investigation of AIH.

Although seeking appropriate animal models to mirror human AIH for *in vivo* studies has been going on for many years, generating such an mouse model fully recapitulated the nosogenesis of AIH in man was deemed to be difficult 39. Con A, a plant lectin isolated from* Canavalia ensiformis* seeds, is capable of driving the recruitment of CD4+T-cells and natural killer T (NKT) cells in the liver by cross-linking the T cell receptors (TCRs) with surface glycoproteins on sinusoidal endothelial cells and major histocompatibility complex (MHC)-II on Kupffer cells 40. Furthermore, increase of serum transaminases, together with the parenchymal cell apoptosis and interface hepatitis in liver, were rapidly induced within several hours upon a single-dose intravenous injection of Con A 41. Thus, Con A-induced hepatitis in mice is regarded as a typical and well-established model for investigating T-cell and macrophage dependent liver injury and mimicking the pathological changes of AIH patients closely 42. For the above reasons, in this study the Con A-induced mouse model of AIH was adopted to elucidate the hepatic differentially expressed miRNA profiling.

Compared to the sham group, 49 differentially expressed miRNAs (31 up-regulated and 18 down-regulated) were detected in the model group (listed in **Supplementary Table 1**). The validation results of the 12 chosen differentially expressed miRNAs with qRT-PCR were in line with the data obtained from the microarray analysis, indicating that our microarray screening data were credible. Another noteworthy part of our present study was that several of these screened miRNAs (e.g. mmu-miR-155, mmu-miR-223, mmu-let-7a) have been reported to be relevant to the pathogenesis of AIH. Suppressors of cytokine signaling (SOCS)-1, a negative regulator of the interleukin (IL)-2 signaling cascade, inhibits the differentiation of both regulatory T cells (Treg) and helper T cells (Th) 17 43, 44. It has been proved that mmu-miR-155 modulated the differentiation of Th17 and Treg by targeting to SOCS1, and the abnormal expression of mmu-miR-155 was considered to be linked to the development of AIH 45. Kupffer cells, the tissue macrophages localized within the liver sinusoid, also contribute to the onset of AIH by producing pro-inflammatory cytokines, presenting antigens to T cells as the antigen-presenting cell (APC), and initiating intra-sinusoidal thrombosis in collaboration with sinusoidal endothelial cells 46. Mmu-miR-223 has been declared to play an important role in the pathogenesis of AIH for inhibiting the expression of IL-1β through absent in melanoma (AIM)-2 pathway and suppressing pro-inflammatory activation of Kupffer cells at the early stage of Con A-induced liver injury 47. As a pleiotropic inflammatory cytokine, IL-6 is essential for the differentiation of Th17 cells 48. Mmu-let-7a was seen as a novel therapeutic strategy in treating AIH for it is able to inhibit the differentiation of Th17 by down-regulating IL-6 secretion 49. Moreover, although the relationships between mmu-miR-210 (another differentially expressed miRNA obtained in our research) and AIH have not been clarified, one study found that mmu-miR-210 has the ability to skew the CD4+Th cell-mediated immune balance in psoriasis (another typical autoimmune disease), characterized by not only inducing the differentiation of Th17 and Th1 cells but also inhibiting the differentiation of Th2 cells, via repressing the expression of signal transducers and activators of transcription (STAT) 6 and LYN (a cytoplasmic membrane-associated tyrosine kinase) 50. In view of the imbalanced differentiation of T lymphocyte subsets is also the hallmark of AIH (including the overactivated Th1 and Th17) 51, it is indicated that miR-210 has a potential ability of participating in the development of AIH. These results taken together showed that some new insights into the pathogenesis of AIH were given to us by opening out the aberrant expression of miRNAs. In addition, the most precursors of these differentially expressed miRNAs expressed on chromosome 2 (shown in **Figure 4**), which was similar to the location of the differentially expressed long non-coding RNAs and differentially expressed mRNAs in the same model (data not shown). These results suggested that the abnormity of chromosome 2 had some kinds of correlations with the development of AIH.

For the primary biological feature for miRNAs is to negatively regulate the target genes by the recognition between miRNA seed regions and mRNA-binding sites, we then predicted the potential targets of these differentially expressed miRNAs with the intersections of two databases: miRWalk and miRDB. There were 959 target genes (601 for up-regulated miRNAs and 358 for down-regulated miRNAs) obtained by the bioinformatic prediction. Through the miRNA-mRNA network analysis, we found each differentially expressed miRNA modulated a range of target genes, and a few of hub genes, which possibly controlled by more than one miRNA, were also revealed from the complicated network. Exploring the biological function of hub genes (e.g. *Cadm1*,* Mier3*) in depth was helpful to gain a better understanding of the complex molecular mechanisms involved in AIH. Furthermore, the complicated regulatory relationships of the mRNA-miRNA pairs were identified and a systematic view for biological function of these miRNAs was acquired through constructing the regulatory networks.

In order to further explore the biological function of these differentially expressed miRNAs and look for the key 'players' in the progress of AIH, the GO analysis was carried out. There were 679 target genes annotated in the 84 GO terms with the threshold of FDR ≤ 0.05. Notably, the target genes of all the differentially expressed miRNAs (including the up-regulated and down-regulated) were significantly involved in BP category of “transcription, DNA-templated” (GO: 0006351), a GO term defined as “the cellular synthesis of RNA on a template of DNA”, emphasizing the regulation feature of these differentially expressed miRNAs in the process of transcription. Moreover, the results of GO analysis also demonstrated that the functions regulated by these differentially expressed miRNAs were not an isolated event, and this regulation was related to the expression balance between these differentially expressed miRNAs. The constructed miRNA-GO-network for the top 5 (in descending sort order of |FC|) up- and down-regulated miRNAs shown that the most significantly regulated GO term in BP categories was “regulation of transcription, DNA-templated” (GO: 0006355), defined as any process that modulates the frequency, rate, or extent of cellular DNA-templated transcription, which also highlighted the regulatory function of these miRNA in regulation of transcription. Meanwhile, mmu-miR-193b-3p contributed more than the other specifically expressed miRNAs to the network (exhibited in 33 GO terms of BP), which implicated its significance in the pathogenesis of AIH.

There were 262 of 959 predicted target genes annotated in 47 KEGG pathways for all differentially expressed miRNAs with the *P* value ≤ 0.05. The top 20 pathways ranked in ascending order by *P*-value were listed in **Figure 9A**, including “Wnt signaling pathway” (path: mmu04310), “Hippo signaling pathway” (path: mmu04390), “Ferroptosis” (path: mmu04216) and “MAPK signaling pathway” (path: mmu04010). The most significantly enriched pathway was “Endocytosis” (path: mmu04144). These results revealed the annotated pathways were the key for the differentially expressed miRNAs involving in the development of AIH. Moreover, our findings also pointed 20 KEGG pathways for the up- (171 of 601 predicted target genes enriched) and 26 KEGG pathways for the down-regulated miRNAs (104 of 385 forecast target genes enriched), and the top 20 pathways ranked in ascending order by *P*-value were also shown in **Table S4 and S5.** There were some common pathways in both **Table S4 and S5**, such as “Wnt signaling pathway”, which signified that the modification of these common signaling pathways resulted from coaction of these screened miRNAs.

Furthermore, the miRNA-pathway-network was also constructed for the top 5 (in descending sort order of |FC|) up- and down-regulated miRNAs which were enriched in the KEGG signaling pathways with the threshold of *P*< 0.05 (shown in **Figure 11**), in order to determine the regulatory relationships between the key miRNAs and hub pathways. In this network, mmu-miR-7005-5p were enriched in 37 pathways, which was more than the other specifically expressed miRNAs. The most significantly enriched pathways were “Endocytosis” (path: mmu04144) and “MAPK signaling pathway”.

Thereafter, the KEGG enrichment was performed to reveal the key signaling pathways that were possibly mediated by these differentially expressed miRNAs. Our findings showed 47 pathways were potentially regulated by these screened miRNAs (threshold set at *P* value <0.05), and the “Endocytosis” pathway was the most significantly enriched one. Endocytosis, defined as a mechanism for cells to remove ligands, nutrients, plasma membrane (PM) proteins, lipids from the cell surface, and bringing them into the cell interior, is crucial in maintenance of immune balance. For example, it is generally known that TCRs, the antigen recognition structures physiologically expressed by all T cells, undergo cycles of endocytosis and recycling, and the balance between these processes ensures the expression and dynamics of TCRs needed for T cells to respond to various antigenic stimuli such that immune responses are efficient and do not cause autoimmunity 52, 53. Thus, it is suggested that “Endocytosis” is an important signal pathway concerning in AIH, even though the exact mechanism is not well understood. Interestingly, except the “Endocytosis” pathway, several other pathways have been previously proven to be correlated with AIH, including “Wnt signaling pathway”, “Hippo signaling pathway”, “Ferroptosis” and “MAPK signaling pathway”. It has been reported that the canonical Wnt/β-catenin signaling pathway in dendritic cells (DCs) favored to maintain the immune homeostasis in intestine and gut, and deficiency of this signaling gave rise to immune responses and lesions in inflammatory bowel disease and colon cancer 54. Akin to the intestine, liver is also an immune exempt organ and the hepatic immune system must remain tolerant, because it is constantly exposed to substantial dietary antigens and microbial products which are of potential immune stimulatory properties 55. Hepatic dendritic cell (HDC), as a unique organ-resident DCs subtype, was the major contributor to sustain tolerance conditions in liver 56.One latest study reported that the deficiency of Wnt signaling pathway in HDCs was found in both AIH patients and the Con A- induced AIH mouse model, and reinvigorating this signaling with a Wnt agonist successfully alleviated the progression of AIH. It is indicated that Wnt signaling pathway in HDCs exerts vital functions on maintenance of the liver homeostasis and their loss altered the immune environment to T cell activation 57. The Hippo signaling pathway, which is evolutionarily conserved, has multiple biological functions in homeostasis and regeneration of tissues or organs. Its core components are Yes-associated protein (YAP) and transcriptional coactivator with PDZ-binding motif (TAZ) 58. Recently, several studies have demonstrated that higher YAP and TAZ expression were found in AIH cases, and the up-regulated expression YAP and TAZ induced the imbalance Treg and Th17 through interacting with retinoid-related orphan receptor gamma (RORγt) and forkhead box P3 (Foxp3) transcription factors 59, 60. Furthermore, the abnormal expression of YAP/TAZ was also connected with the fibrotic process in chronic hepatitis patients, including AIH patients 61. Iron deposition can be observed in autoinflammatory diseases frequently, and promote the expression of proinflammatory cytokines in T cells, including granulocyte-macrophage colony-stimulating factor (GM-CSF) and IL-2, via regulating the stability of poly(rC)-binding protein 1 (PCBP1) 62. Accumulation of iron give rise to lethal lipid peroxidation, which was known as a new regulated cells death manner named ferroptosis 63. It was recently found out that the excessive nitrogen stress response associated ferroptosis indeed participated in Con A-induced AIH, which was a pivotal step that drives the execution of immune-mediated hepatic damage, and the precise mechanism of Caveolin-1 (Cav-1) on protecting hepatocytes in AIH was linked to against the reactive nitrogen species (RNS)- mediated ferroptosis 64. Concerning the MAPK signaling pathway, more and more evidences reveal the essential role which it played in the pathophysiological processes of inflammation in liver 65. Various proinflammatory cytokines, comprising interferon (IFN)-γ, tumor necrosis factor (TNF)-α, IL-1β, IL-2, IL-6 and IL-12, act a pivotal role in AIH 66. It has been reported that MAPK signaling pathway is closely related to regulate the cytokines network, and inhibiting the activation of MAPK signaling pathway in some ways was reputed to be a promising candidate for therapy of AIH 67, 68. Our present findings not only corroborated in the sense that these signaling pathways which have been previously implicated in AIH were possibly regulated by these 49 miRNAs, but also were conducive to gain a deeper understanding how these pathways functioned in AIH. In addition, combining with other relevant studies, the other enriched pathways which have not yet been credibly reported (e.g. endocytosis) are also likely to be involved in the not well clarified mechanisms of AIH and serve as the promising candidate therapeutic targets for this disease.

## Conclusion

In summary, the profiling of differentially expressed miRNAs in Con A-induced AIH mouse was screened for the first time in our current study, and a set of 49 aberrantly expressed miRNAs, which bear the potential to be the molecular markers referring to the onset of AIH, were identified. The target genes of these miRNAs were also predicted with bioinformatics analysis, and a much more comprehensive analysis of these target genes provided novel information contributing to a better understanding of the molecular mechanisms as well as biological pathways implicated in AIH. Moreover, numerous hub genes, which probably controlled by several miRNAs, were gained, and intensive study of these hub genes will consolidate our realization on the involvement of miRNAs in the pathophysiology of AIH. Altogether, although further mechanism research is still required, our findings presented herein not only provide novel insights into the pathogenesis of AIH, but also suggest that miRNA profiling is a strategy with great application prospect to be applied in the diagnosis and better management of this disease.

## Supplementary Material

Supplementary tables.

## Figures and Tables

**Table 1:** Features of the miRNAs selected for qRT-PCR validation

miRNA	Number of mirbase accession	Fold Change	*P-*Value	Regulation
mmu-miR-21a-3p	MIMAT0004628	42.42	2.60E-06	up
mmu-miR-1934-3p	MIMAT0017341	35.46	3.60E-08	up
mmu-miR-188-5p	MIMAT0000217	34.71	4.18E-03	up
mmu-miR-7005-5p	MIMAT0027914	31.75	2.99E-04	up
mmu-miR-3081-5p	MIMAT0014870	24.56	2.16E-02	up
mmu-miR-2861	MIMAT0013803	22.57	5.90E-03	up
mmu-miR-126a-5p	MIMAT0000137	-6.19	3.67E-02	down
mmu-miR-7055-3p	MIMAT0028015	-6.37	6.83E-03	down
mmu-miR-3058-5p	MIMAT0014813	-6.43	1.34E-02	down
mmu-miR-22-5p	MIMAT0004629	-7.00	9.61E-03	down
mmu-miR-7080-3p	MIMAT0028067	-7.02	5.64E-03	down
mmu-miR-193b-3p	MIMAT0004859	-8.30	1.38E-02	down

**Table 2:** The sequences of primers used in qRT-PCR experiments

miRNA	Primers
mmu-miR-21a-3p	RT: 5'-CTCAACTGGTGTCGTGGAGTCGGCAATTCAGTTGAGGACAGCCC-3';
	F: 5'-ACACTCCAGCTGGGCAACAGCAGTCGAT-3'.
mmu-miR-1934-3p	RT: 5'-CTCAACTGGTGTCGTGGAGTCGGCAATTCAGTTGAGTCACCAGC-3';
	F: 5'-ACACTCCAGCTGGGAGGATGACGGTGGGG-3'.
mmu-miR-188-5p	RT: 5'-CTCAACTGGTGTCGTGGAGTCGGCAATTCAGTTGAGCCCTCCA-3';
	F: 5'-ACACTCCAGCTGGGCATCCCTTGCATGG-3'.
mmu-miR-7005-5p	RT: 5'-CTCAACTGGTGTCGTGGAGTCGGCAATTCAGTTGAGTGCTGGTC-3';
	F: 5'-ACACTCCAGCTGGGCCTGGGGATGGGAGG-3'.
mmu-miR-3081-5p	RT; 5'-CTCAACTGGTGTCGTGGAGTCGGCAATTCAGTTGAGGCTCACCG-3';
	F: 5'-ACACTCCAGCTGGGGACTGGAGCTTGGAG-3'.
mmu-miR-2861	RT: 5'-CTCAACTGGTGTCGTGGAGTCGGCAATTCAGTTGAGCCGCCCG-3';
	F: 5'-ACACTCCAGCTGGGGGGGCCTGGCGGC-3'.
mmu-miR-126a-5p	RT: 5'-CTCAACTGGTGTCGTGGAGTCGGCAATTCAGTTGAGCGCGTAC-3';
	F: 5'-ACACTCCAGCTGGGCATTATTACTTTTGG-3'.
mmu-miR-7055-3p	RT: 5'-CTCAACTGGTGTCGTGGAGTCGGCAATTCAGTTGAGCTGGTGGG-3';
	F: 5'-ACACTCCAGCTGGGTTGCTACTTTGATAC-3'.
mmu-miR-3058-5p	RT: 5'-CTCAACTGGTGTCGTGGAGTCGGCAATTCAGTTGAGTCTTCCAG-3';
	F: 5'-ACACTCCAGCTGGGTCAGCCACGGCTTAC-3'.
mmu-miR-22-5p	RT: 5'-CTCAACTGGTGTCGTGGAGTCGGCAATTCAGTTGAGTAAAGCTT-3';
	F: 5'-ACACTCCAGCTGGGAGTTCTTCAGTGGC-3'.
mmu-miR-7080-3p	RT: 5'-CTCAACTGGTGTCGTGGAGTCGGCAATTCAGTTGAGAGGGAACG-3';
	F: 5'-ACACTCCAGCTGGGCAGGCTCACCCCTC-3'.
mmu-miR-193b-3p	RT: 5'-CTCAACTGGTGTCGTGGAGTCGGCAATTCAGTTGAGAGCGGGAC-3';
	F: 5'-ACACTCCAGCTGGGAACTGGCCCACAAA-3'.
All	R: 5'-TGGTGTCGTGGAGTCG-3'.
U6	F: 5'-CTCGCTTCGGCAGCACA-3';
	R: 5'-AACGCTTCACGAATTTGCGT-3'.

## Abbreviations

AIH: autoimmune hepatitis
IgG: immunoglobulin G
ALT: alanine aminotransferase
AST: aspartate aminotransferase
miRNAs: microRNAs
mRNA: messenger RNA
AGO: Argonaut proteins
UTR: untranslated region
Con A: concanavalin A
SPF: specific pathogen free
PCA: principal component analysis
FC: fold change
qRT-PCR: quantitative real-time polymerase chain reaction
GO: Gene ontology
KEGG: Kyoto Encyclopedia of Genes and Genomes
CC: cellular components
MF: molecular functions
BP: biological processes
FDR: false discovery rate
SPSS: Statistic Package for Social Science
lncRNA: long non-coding RNA
NKT: natural killer T
TCR: T cell receptor
MHC: major histocompatibility complex
SOCS: suppressors of cytokine signaling
IL: interleukin
Treg: regulatory T cells
Th: helper T cell
APC: antigen-presenting cells
AIM: absent in melanoma
STAT: signal transducers and activators of transcription
PM: plasma membrane
MAPK: mitogen-activated protein kinase
DC: dendritic cell
HDC: hepatic dendritic cell
YAP: yes-associated protein
TAZ: transcriptional coactivator with PDZ-binding motif
RORγt: retinoid-related orphan receptor gamma
Foxp3: forkhead box P3
GM-CSF: granulocyte-macrophage colony-stimulating factor
PCBP1: poly(rC)-binding protein 1
Cav-1: Caveolin-1
RNS: reactive nitrogen species
IFN-γ: interferon-γ
TNF-α: tumor necrosis factor-α
