# Supplementary material for: Characterization and functional prediction of the microRNAs differentially expressed in a mouse model of concanavalin A-induced autoimmune hepatitis
Source: Int J Med Sci. 2020 Aug 27;17(15):2312–27. doi: 10.7150/ijms.47766 (PMC7484648; doi:10.7150/ijms.47766)
Supplement: Supplementary file 1 — Supplementary tables. [file ijmsv17p2312s1.pdf]

Table S1. Features of the 49 differentially expressed miRNAs

miRNA	Number of mirbase accession	Fold Change	*P*-Value	Regulation
mmu-miR-3091-5p	MIMAT0014903	71.90	2.04E-06	up
mmu-miR-6538	MIMAT0025583	69.76	2.70E-03	up
mmu-miR-1897-5p	MIMAT0007864	54.79	2.22E-05	up
mmu-miR-5128	MIMAT0020639	43.03	1.75E-03	up
mmu-miR-21a-3p	MIMAT0004628	42.42	2.60E-06	up
mmu-miR-1934-3p	MIMAT0017341	35.46	3.60E-08	up
mmu-miR-188-5p	MIMAT0000217	34.71	4.18E-03	up
mmu-miR-7005-5p	MIMAT0027914	31.75	2.99E-04	up
mmu-miR-3081-5p	MIMAT0014870	24.56	2.16E-02	up
mmu-miR-2861	MIMAT0013803	22.57	5.90E-03	up
mmu-miR-3473f	MIMAT0031390	22.49	4.31E-02	up
mmu-miR-155-5p	MIMAT0000165	20.66	2.96E-03	up
mmu-miR-125a-3p	MIMAT0004528	18.25	7.46E-06	up
mmu-miR-3470a	MIMAT0015640	15.88	4.69E-02	up
mmu-miR-18a-5p	MIMAT0000528	10.57	1.02E-03	up
mmu-miR-7002-5p	MIMAT0027906	10.31	2.26E-02	up
mmu-miR-721	MIMAT0003515	9.70	1.78E-02	up
mmu-miR-877-5p	MIMAT0004861	7.27	8.23E-03	up
mmu-miR-6931-5p	MIMAT0027762	5.63	1.31E-02	up
mmu-miR-3102-5p	MIMAT0014933	4.62	3.84E-05	up
mmu-miR-135a-1-3p	MIMAT0004531	4.54	4.60E-02	up
mmu-miR-5126	MIMAT0020637	3.71	3.60E-05	up
mmu-miR-211-3p	MIMAT0017059	3.68	1.02E-04	up
mmu-miR-3960	MIMAT0019336	3.63	9.13E-05	up
mmu-miR-8101	MIMAT0031405	3.40	3.10E-03	up

mmu-miR-486a-5p	MIMAT0003130	2.78	2.45E-02	up
mmu-miR-223-3p	MIMAT0000665	2.62	5.59E-04	up
mmu-miR-210-3p	MIMAT0000658	2.55	9.68E-03	up
mmu-miR-8110	MIMAT0031416	2.38	8.03E-04	up
mmu-miR-451a	MIMAT0001632	2.26	2.04E-02	up
mmu-miR-2137	MIMAT0011213	2.25	4.84E-03	up
mmu-miR-101c	MIMAT0019349	-2.06	1.55E-03	down
mmu-miR-712-5p	MIMAT0003502	-2.44	3.07E-02	down
mmu-miR-3475-5p	MIMAT0026642	-3.30	4.26E-02	down
mmu-miR-129b-5p	MIMAT0029862	-4.25	2.55E-02	down
mmu-miR-19a-3p	MIMAT0000651	-4.30	2.63E-02	down
mmu-miR-378a-5p	MIMAT0000742	-4.70	2.48E-02	down
mmu-miR-532-5p	MIMAT0002889	-4.96	2.93E-02	down
mmu-miR-877-3p	MIMAT0004862	-5.55	2.47E-02	down
mmu-miR-126a-5p	MIMAT0000137	-6.19	3.67E-02	down
mmu-miR-7055-3p	MIMAT0028015	-6.37	6.83E-03	down
mmu-miR-3058-5p	MIMAT0014813	-6.43	1.34E-02	down
mmu-miR-22-5p	MIMAT0004629	-7.00	9.61E-03	down
mmu-miR-7080-3p	MIMAT0028067	-7.02	5.64E-03	down
mmu-miR-193b-3p	MIMAT0004859	-8.30	1.38E-02	down
mmu-miR-7046-3p	MIMAT0027997	-8.46	1.45E-02	down
mmu-let-7f-1-3p	MIMAT0004623	-8.67	1.39E-02	down
mmu-miR-802-5p	MIMAT0004188	-13.36	1.99E-05	down
mmu-miR-1927	MIMAT0009390	-34.06	1.97E-06	down

Table S2. GO enrichment analysis for the up-regulated miRNAs (Top 30)

GO terms	GeneRatio	*P* value	Category
protein binding	0.362997658	7.59E-09	MF
transcription, DNA-templated	0.16937355	2.36E-07	BP
negative regulation of transcription from RNA polymerase II promoter	0.090487239	1.14E-06	BP
DNA binding	0.156908665	1.47E-06	MF
neuronal cell body	0.06993007	5.99E-06	BP
regulation of clathrin-dependent endocytosis	0.009280742	1.33E-05	BP
metal ion binding	0.224824356	1.92E-05	MF
regulation of ARF protein signal transduction	0.016241299	1.94E-05	BP
ion channel binding	0.028103044	2.64E-05	MF
positive regulation of transcription from RNA polymerase II promoter	0.102088167	4.15E-05	BP
regulation of transcription, DNA-templated	0.167053364	4.99E-05	BP
RNA polymerase II regulatory region sequence-specific DNA binding	0.042154567	5.08E-05	MF
regulation of heart rate by cardiac conduction	0.013921114	5.15E-05	BP
positive regulation of transcription, DNA-templated	0.067285383	5.54E-05	BP

negative regulation of transcription, DNA-templated	0.05800464	1.03E-04	BP
membrane	0.400932401	1.29E-04	BP
nucleus	0.365967366	2.78E-04	BP
potassium ion homeostasis	0.009280742	3.03E-04	BP
clathrin coat	0.006993007	3.07E-04	BP
transcriptional activator activity, RNA polymerase II transcription regulatory region sequence-specific DNA binding	0.025761124	3.62E-04	MF
DNA binding transcription factor activity	0.086651054	3.69E-04	MF
chromatin binding	0.053864169	3.88E-04	MF
nervous system development	0.048723898	4.26E-04	BP
nucleotide binding	0.131147541	4.40E-04	MF
core promoter sequence-specific DNA binding	0.014051522	4.65E-04	MF
cell volume homeostasis	0.009280742	4.92E-04	BP
sequence-specific double-stranded DNA binding	0.007025761	4.98E-04	MF
potassium ion transport	0.023201856	5.10E-04	BP
voltage-gated ion channel activity	0.023419204	6.09E-04	MF
postsynaptic density	0.032634033	6.13E-04	BP

Table S3. GO enrichment analysis for the down-regulated miRNAs (Top 30)

GO terms	GeneRatio	*P* value	Category
transcription, DNA-templated	0.238267148	3.56E-13	BP
regulation of transcription, DNA-templated	0.245487365	1.64E-11	BP
positive regulation of transcription, DNA-templated	0.111913357	3.90E-10	BP
nucleoplasm	0.220216606	4.30E-10	CC
nucleus	0.458483755	1.20E-09	CC
DNA binding	0.200729927	3.17E-09	MF
positive regulation of transcription from RNA polymerase II promoter	0.144404332	1.42E-08	BP
RNA polymerase II proximal promoter sequence-specific DNA binding	0.072992701	2.49E-06	MF
sequence-specific DNA binding	0.091240876	3.69E-06	MF
chromatin organization	0.057761733	9.61E-06	BP
nucleic acid binding	0.102189781	1.22E-05	MF
protein binding	0.354014599	1.51E-05	MF
chromatin binding	0.072992701	1.54E-05	MF
cytoplasm	0.440433213	2.16E-05	CC

negative regulation of transcription from RNA polymerase II promoter	0.093862816	3.67E-05	BP
long-chain fatty acid biosynthetic process	0.010830325	4.94E-05	BP
transcriptional activator activity, RNA polymerase II proximal promoter sequence-specific DNA binding	0.051094891	5.78E-05	MF
regulation of transcription from RNA polymerase II promoter	0.068592058	7.72E-05	BP
metal ion binding	0.237226277	7.83E-05	MF
methylated histone binding	0.02189781	8.32E-05	MF
hair follicle maturation	0.010830325	8.56E-05	BP
DNA binding transcription factor activity	0.102189781	1.33E-04	MF
poly(G) binding	0.010948905	1.35E-04	MF
poly(U) RNA binding	0.01459854	1.40E-04	MF
neuron projection	0.064981949	2.61E-04	CC
zinc ion binding	0.076642336	2.85E-04	MF
neuron development	0.02166065	2.87E-04	BP
neuron differentiation	0.028880866	4.11E-04	BP
macromolecular complex	0.083032491	4.31E-04	CC
central nervous system neuron differentiation	0.010830325	5.11E-04	BP

Table S4. KEGG enrichment analysis for the up-regulated miRNAs (Top 20)

KEGG pathways	ListHit	GeneRatio	*P* value
Endocytosis	15	0.087719298	4.32E-04
Calcium signaling pathway	11	0.064327485	1.98E-03
Prolactin signaling pathway	6	0.035087719	4.59E-03
Oxytocin signaling pathway	9	0.052631579	5.50E-03
Regulation of actin cytoskeleton	11	0.064327485	5.93E-03
Phosphatidylinositol signaling system	6	0.035087719	1.96E-02
Inositol phosphate metabolism	5	0.029239766	1.98E-02
Circadian rhythm	3	0.01754386	2.66E-02
Endocrine and other factor-regulated calcium reabsorption	4	0.023391813	2.93E-02
Signaling pathways regulating pluripotency of stem cells	7	0.040935673	2.94E-02
Purine metabolism	8	0.046783626	3.35E-02
Axon guidance	8	0.046783626	3.66E-02
GABAergic synapse	5	0.029239766	4.04E-02
Breast cancer	7	0.040935673	4.07E-02

Wnt signaling pathway	7	0.040935673	4.07E-02
Glycerolipid metabolism	4	0.023391813	4.09E-02
Dilated cardiomyopathy (DCM)	5	0.029239766	4.22E-02
Morphine addiction	5	0.029239766	4.39E-02
GnRH signaling pathway	5	0.029239766	4.39E-02
mTOR signaling pathway	7	0.040935673	4.74E-02

Table S5. KEGG enrichment analysis for the down-regulated miRNAs (Top 20)

KEGG pathways	ListHit	GeneRatio	*P* value
Endocrine and other factor-regulated calcium reabsorption	5	0.048076923	6.91E-04
Long-term depression	5	0.048076923	1.04E-03
Ferroptosis	4	0.038461538	1.82E-03
MicroRNAs in cancer	7	0.067307692	2.65E-03
Hippo signaling pathway	7	0.067307692	4.01E-03
Thyroid hormone signaling pathway	6	0.057692308	4.05E-03
Gap junction	5	0.048076923	4.94E-03
Insulin signaling pathway	6	0.057692308	1.00E-02
Wnt signaling pathway	6	0.057692308	1.30E-02
Aldosterone-regulated sodium reabsorption	3	0.028846154	1.35E-02
Fatty acid biosynthesis	2	0.019230769	1.42E-02
Adipocytokine signaling pathway	4	0.038461538	1.42E-02
cAMP signaling pathway	7	0.067307692	1.48E-02
Prolactin signaling pathway	4	0.038461538	1.49E-02

Hedgehog signaling pathway	3	0.028846154	1.77E-02
Salivary secretion	4	0.038461538	1.78E-02
Morphine addiction	4	0.038461538	2.98E-02
GnRH signaling pathway	4	0.038461538	2.98E-02
Mannose type O-glycan biosynthesis	2	0.019230769	3.08E-02
Progesterone-mediated oocyte maturation	4	0.038461538	3.09E-02
